# Polymethylolacrylamide/AuNPs Nanocomposites: Electrochemical Synthesis and Functional Characteristics

**DOI:** 10.3390/polym13142382

**Published:** 2021-07-20

**Authors:** Lidiia Kolzunova, Elena Shchitovskaya, Maxim Karpenko

**Affiliations:** 1 Institute of Chemistry, Far East Branch of the Russian Academy of Sciences, 100_letiya Vladivostoka pr. 159, Vladivostok 690022, Russia; akm@ich.dvo.ru; 2 Department of Physical and Analytical Chemistry, School of Natural Sciences, Campus, 10 Ajax Bay, Russky Island, Far Eastern Federal University (FEFU), Vladivostok 690922, Russia

**Keywords:** composite polymer-gold nanoparticles, electropolymerization, electrocatalytic and sensory properties

## Abstract

In this study the advantages of the electrochemical approach to the formation of polymer/metal nanoparticle composites are demonstrated. The method enables one to simplify the multistage processes of traditional technologies for the production of such materials through combining all intermediate processes in one stage and reducing the total formation time to 3–10 min. The possibility of a single-stage formation of a polymethylolacrylamide/AuNPs composite through including AuNPs into an electrically non-conducting polymethylolacrylamide film (carrier) formed by electropolymerization through potentiostatic electrolysis is also demonstrated for the first time. It is established that the addition of tetrachloroauric acid (HAuCl_4_·4H_2_O) into a monomeric composition containing acrylamide, formaldehyde, N,N′-methylene-bis-acrylamide, zinc chloride, and H_2_O results in simultaneous electrochemical initiation of polymerization with the formation of a polymer film on the cathode, electrolytic reduction of gold ions to Au^0^, and immobilization of AuNPs particles into the growing polymer matrix. It was found that the formation of the PMAA / AuNPs composite is energetically more favorable than the synthesis of the main PMAA film, since it proceeds at a lower cathodic potential. The inclusion of AuNPs into the polymethylolacrylamide film was confirmed visually, as well as by X-ray phase analysis, small-angle X-ray scattering, microscopy, and element analysis. The gold content in the composite increases along with the increase of the concentration of HAuCl_4_ in the electrolyte. The radius of the AuNPs particles was found to range between 3 and 7 nm. The AuNPs particles are spherical in shape and can combine into larger clusters containing up to 10 or more particles. The dynamics of formation, structure, and morphology of the polymethylolacrylamide/AuNPs composite were investigated. It was revealed that gold nanoparticles are mainly concentrated in the near-electrode and near-solution layers of the composite. We found that the composite has electrocatalytic activity. The possibility of its use as a sensor for hydrogen peroxide is demonstrated.

## 1. Introduction

One of the most urgent areas of advanced chemistry consists in the creation of composites with an active component in the form of nano- and microcrystals distributed over the bulk of a polymer matrix, which stabilizes them, thus maintaining high activity of nanoparticles and remaining a highly elastic carrier. Such composites, consisting of two or more components that differ in chemical composition and are separated by a pronounced boundary, combine the properties of a polymer and a metal or acquire new qualities. This new class of materials is extensively applied in various technologies: information and computers [1], ecology [2], chemistry, and biology [3,4,5]. These materials are used to create highly sensitive selective electrodes and various types of sensors [3,6,7,8,9,10,11], catalysts and electrocatalysts [7,12], and medicine [9,13]. Nanoparticles on a carrier provide an increased sensitivity due to a larger specific surface area as compared to conventional catalysts and sensors based on dispersed metals. The use of such composites successfully resolves the tasks of simultaneous deoxygenation, softening, and desalination of water and the removal of microorganisms from it [14,15]. Organometallic composites, due to their high specific surface area and adjustable porosity, can serve as excellent sensors for quantifying the relative humidity of the air [2]. Here, due to the protective function of a polymer, metal nanoparticles in composite electrodes are less exposed to corrosion and surface passivation as compared to solid metal, as a result of which the reproducibility of the results is higher, and the service life of such electrodes is longer than that of all-metal ones.

Nanoparticles of Cu [13,14,15], Ni, Co, and Fe [5] and metal oxides (SnO_2_, Fe_3_O_4_) [16,17] are used as fillers of a polymer matrix. However, the most interesting materials here are nanocomposite materials with the inclusion of noble metal particles: Ag [18,19,20], Au [6,21,22,23,24,25,26,27,28,29,30], Pt [30,31,32], and Pd [19]. Most published studies on this subject are devoted to the production and application of hybrid materials containing AuNPs. This is related to the fact that gold nanoparticles are in demand in such areas as medicine and nanomedicine, biology, chemistry, physics, various biotechnologies, biosensors, catalysis, and analytical chemistry [28].

To create polymer/metal nanoparticle hybrid materials, the following methods are traditionally used: (1) separate synthesis of a polymer and metal nanoparticles, followed by their inclusion into the polymer matrix; (2) synthesis of a polymer, its impregnation with metal salts with subsequent reduction to metal by chemical [3,14,15,25,26,27,28,29], physical [22], or electrochemical [4,13,23,30,31] methods; (3) synthesis of metal nanoparticles and their addition to a monomer composition for polymerization; (4) synthesis of a polymer with subsequent deposition of a metal on its surface. All of these technologies are complex, multi-stage (≥3 stages), and time-consuming. Therefore, there is a constant search for simpler methods of forming polymer nanocomposites.

The electrochemical approach seems to be the most promising from the point of view of simplifying the technology of forming polymer/metal nanoparticle composites. It enables one not only to reduce the number of intermediate steps to 1–2 [23,30,31], but, in some cases, even to combine in one process the synthesis of a polymer by electropolymerization, the electro-reduction of metal ions to Me^0^, and the immobilization of metal nanoparticles MeNPs in a growing polymer matrix [4,13,32,33].

The method of electropolymerization allows fabrication of both electrically conducting and electroneutral polymers. An analysis of the published works showed an interest in hybrids based on conductive polymers (CP) [19]. Numerous charged functional groups in the main chains of CP make them perfect facilitators for including various metals into a composite. Aniline, pyrrole, thiophene and their derivatives [13,19,23,33], aminophenols [30], dopamine [4], diphenylamine [31], and others are used as monomers in such processes. However, it must be taken into account that electrically conducting polymers can chemically interact with metal ions, thus reducing the amount of free MeNPs, which results in a deterioration of the catalytic or sensory properties of a composite material. Therefore, it is evident that the use of an inert polymer matrix is preferable [20,32]. However, there are very few works on the matter, so that such studies are of great urgency.

Within the scope of the present work, we studied the possibility of a single-stage formation of a polymethylolacrylamide/gold nanoparticle composite by including AuNPs into an electrically non-conducting polymethylolacrylamide film (carrier) formed by electropolymerization. The study was aimed at simplifying the technology of forming metal/polymer composite materials and expanding their range, as well as on broadening theoretical and experimental knowledge about the electrochemical synthesis mechanism.

## 2. Materials and Methods

### 2.1. Solutions for the Formation of Composites

#### 2.1.1. Chemicals

The following substances were used to form metal-polymer composites. Acrylamide (AA) 2-stage crystallizing (Paneco, Moscow, Russia), N,N′-methylene-bis-acrylamide (MBAA) 98 % (Paneco, Moscow, Russia), formaldehyde (F) 29–31 % Russian State Standard No. 1625-89 (Neva Reaktiv, St. Petersburg, Russia), HAuCl_4_∙4H_2_O of the pure grade (Voikov factory, Moscow, Russia), hydrochloric acid of the chemically pure grade (Neva Reaktiv, St. Petersburg, Russia), phosphate buffer pH 6.86 (PBS), (Uralkhiminvest, Ufa, Russia), and zinc chloride (Scharlau, Sentmenat, Spain).

Zinc chloride was used as a 2–4 mol/L solution, pH 3–4. The exact concentration of zinc chloride was determined by complexometric titration with Trilon B.

Chitosan chloride (Chs) (ICH FEB RAS, Vladivostok, Russia) of a degree of deacylation of 0.7 and MW = 4.4 × 10^5^ Da was used as a 1–2% solution.

#### 2.1.2. Electrolytes

The compositions of electrolytes used for electropolymerization are provided in Table 1. We chose such a monomeric composition in accordance with our preliminary research [34]. In that scientific publication, we studied in detail the effect of the concentration of acrylamide, formaldehyde, and zinc chloride, as well as the ratio of the concentrations of monomers on the speed and quality of the polymer film formed by the method of electropolymerization. It was found that the optimum is the molar ratio AA:F = 1:1 and the concentration of the main components C_AA_ = 3 mol/L, C_F_ = 3 mol/L and C_ZnCl2_ = 0.2 mol/L.

Solutions for the synthesis of composites were prepared immediately before the start of the experiment. Deionized water was used as a solvent.

### 2.2. Electrochemical Synthesis of the Polymethylolacrylamide/AuNPs Composites

The synthesis and study of the electrochemical behavior of the composites was carried out using a Solartron 1287A (Solartron Analytical UK, Farnborough, Great Britain) and an IPC-Pro 8.0 (Cronas, Moscow, Russia) potentiostat/galvanostat with access to a personal computer. The cell was connected to a potentiostat using a three-electrode circuit.

**Figure 1:**
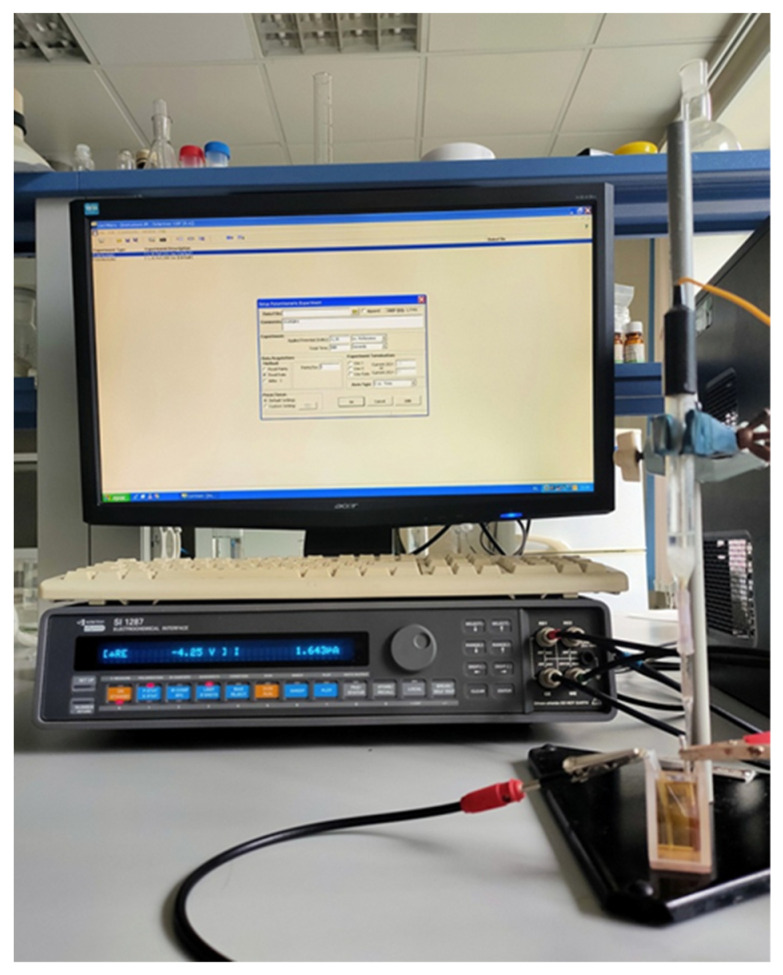
The equipment for the electrochemical syntheses.

Electropolymerization was carried out in glass cells of a volume of 10 cm^3^ without separation of the cathode and anode space and without degassing. The composites were formed at room temperature. The cathode potential was (−1.16 ÷ −1.2) V. The electrolysis time was 1–5 min. The equipment for the electrochemical syntheses is shown in Figure 1.

The working electrodes (cathode) were made of AISI 304 and 12X18H10 stainless steel rods (S = 1–1.5 cm^2^). The steel rods were polished, degreased with a paste of fine magnesium oxide, and thoroughly washed with deionized water. A platinum plate of S = 7.5 cm^2^ was used as an auxiliary electrode (anode). The reference electrode was a saturated silver chloride electrode EVL-1M3 equipped with a Luggin capillary.

To separate the composite film of polymethylolacrylamide/AuNPs (PMAA/AuNPs) from the steel substrate, the coated electrode was immersed in 0.1–0.5 mol/L HCl for 90–100 s. As a result of the dissolution of a thin sublayer of metallic zinc reduced at the cathode during electrolysis, the film lost its adhesion to the substrate and was easily separated from it.

### 2.3. Swelling Capacity of the Composite Films

The swelling capacity (*S_sw_*) of the films in water was calculated by the equation:

(1) $$S_{sw}=\left[\frac{m_{sw}-m_{dry}}{m_{dry}}\right]\times 100\%$$

where *m_sw_* is the weight of a swollen film, gram, and *m_dry_* is the weight of a dry film, gram.

### 2.4. Coefficients of Linear Extension/Compression of the Films

The coefficients of linear extension *L*_1_ during the swelling in water and linear compression (shortening) *L*_2_ during the drying of the polymer films and composites were determined after separation of the composite film from the electrode. The values of *L*_1_ and *L*_2_ were calculated using the equations:

(2) $$L_{1}=\frac{{Ø}_{sw}}{{Ø}_{in}};\;\;\;\;\;L_{2}=\frac{{Ø}_{in}}{{Ø}_{dry}}$$

where *Ø_sw_* is the diameter of the swollen film, *Ø_in_* is the initial diameter of the film, *Q_dry_* is the diameter of the dried film, *L*_1_ is the linear extension coefficient of the electrosynthesized film, and *L*_2_ is the linear shortening coefficient of the electrosynthesized film.

### 2.5. Research Methods

The structure and morphology of the polymer films and the local content of gold immobilized in the polymer film were studied using a PHENOM pro-X electron microscope (Thermo Fisher Scientific, Waltham, MA, USA), and a Zeiss Libra-200 FE transmission electron microscope (Carl Zeiss, Oberkochen, Germany). Thin cross-sections of the composite film were examined using an Axioplan 2 Imaging optical microscope (Carl Zeiss, Mannheim, Germany) with a Sony NV-GS330EE-S (Panasonic, Osaka, Japan) photographic accessory.

The X-ray diffraction (XRD) patterns of the composite films were recorded using a D_8_ ADVANCE X-ray diffractometer (Bruker, Karlsruhe, Germany) in the CuKα-radiation. The EVA search program with the PDF-2 database (Powder Diffraction File; Kabekkodu, 2007) was used for the X-ray patterns processing.

The size distribution of gold particles was estimated by the small angle X-ray scattering (SAXS) on a HECUS S3-MICRO-PIX diffractometer (Hecus X-Ray Systems, Graz, Austria).

## 3. Results and Discussion

The most studied compositions for the formation of polymer coatings and films on metals by electropolymerization are aqueous solutions of acrylamide and its derivatives containing an additive of ZnCl_2_ as an indirect initiator of polymerization [34]. From such solutions (Table 1, electrolytes 1 and 2), a uniform film of polymethylolacrylamide [-CH_2_-CH-(CONH-CH_2_OH)-]_n_ is formed on the cathode during the electrolysis. A distinctive feature of the electrochemically synthesized polymethylolacrylamide (PMAA) consists in its porous structure [35], which allows the introduction of metal nanoparticles into the PMAA film without destroying the polymer matrix. In addition, such a polymer is non-electrically conducting [32], which excludes direct reduction of metals on the PMAA surface, and does not contain polar functional groups capable of interacting with other components of the solution. These characteristics make it possible to introduce metal nanoparticles into the PMAA film without chemical binding to it and to obtain composites with a clearly defined polymer/metal interface.

In the present work, the possibility of a single-stage electrochemical formation of a composite based on polymethylolacrylamide with the immobilization of gold nanoparticles into a polymer matrix was investigated. The electrolytes 1 and 2 (Table 1) were used as base solutions, then tetrachloroauric acid HAuCl_4_·4H_2_O was introduced into them (Table 1, electrolytes 3 and 4).

### 3.1. Mechanism of Reduction of [AuCl_4_]^−^ and Formation of the PMAA/AuNPs Composite

In [32], using the example of the synthesis of a composite based on polymethylolacrylamide with the inclusion of platinum nanoparticles, we showed that the electropolymerization reaction was one of the few that allows combining a number of successive stages in one process: (1) the formation of active particles and the initiation of polymerization, (2) the formation of a polymer film, (3) the electro-reduction of metal ions to Me^0^, and (4) the immobilization of Me^0^ into a growing polymer matrix. However, one should take into account that such a one-step synthesis is possible only if: (1) the polymerization initiation potential and the metal reduction one are similar, or the metal is reduced at a lower cathode potential, and (2) the components of the monomer composition do not chemically interact with the salt of the introduced metal. Therefore, first of all, it was crucial to find out whether the above conditions are met for electrolytes 3 and 4 (Table 1).

#### 3.1.1. Possibility of Chemical Reduction of a Tetrachloroaurate Ion [AuCl_4_]^−^

As follows from Table 1, all the electrolytes for the formation of polymer films and composites based on them contain formaldehyde. Formaldehyde is known to be one of the organic reducing agents that are used to extract gold from solutions with a low concentration [36]. The reduction process goes on better in neutral and alkaline media. The resulting gold-containing solution has a cherry-red color. Based on the above information, first of all, it was necessary to determine whether the chemical reduction of gold occurs in the electrolytes 3 and 4, which contain both tetrachloroauric acid and formaldehyde (pH ~3).

**Figure 2:**
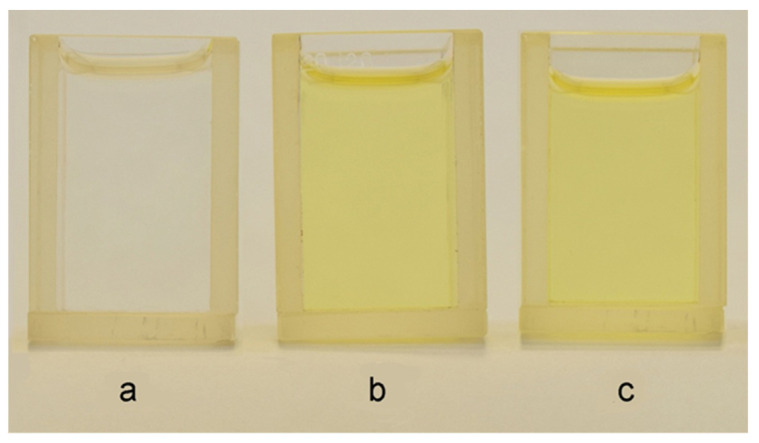
Color of solutions: electrolyte 2 (**a**), electrolyte 3 (**b**), electrolyte 3 after 60 min (**c**).

The initial solutions of monomers for electropolymerization (Table 1, electrolytes 1 and 2) are colorless and transparent (Figure 2a). After the introduction of HAuCl_4_, they acquire the yellow color typical for solutions of tetrachloroauric acid (Figure 2b). It was found that this color did not change, at least for 60 min after the preparation of the solution (Figure 2c), despite the fact that the electrolyte contained such a strong reducing agent as formaldehyde.

**Figure 3:**
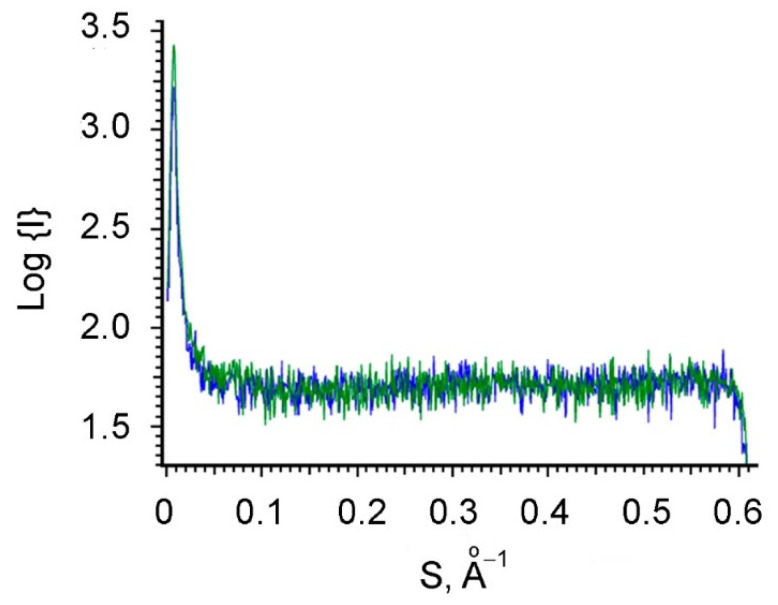
Small-angle X-ray spectrum of electrolytes 3 and 4. The holding time of the solutions is 0–60 min.

Further investigation by means of the small angle X-ray scattering (SAXS) of the electrolytes 3 and 4 containing HAuCl_4_ and formaldehyde showed the absence of any nanoparticles both in freshly prepared solutions and after 60 min of exposure. This is indicated by the fact that the intensity curves of both solutions are identical and represent an almost straight line (Figure 3), which does not enable us to build a spectrum of the particle size distribution.

The analysis of the obtained results enables us to conclude that, in the studied water-monomer compositions containing the additive of tetrachloroauric acid, there is no chemical reduction of [AuCl_4_]^−^ ions to AuNPs by formaldehyde.

#### 3.1.2. Electrochemical Reduction of a Tetrachloraurate Ion [AuCl_4_]^−^

Further research was aimed at studying the features of the electrochemical reduction of gold at the potential of electropolymerization of monomers. According to the published data [37], the electrolytic reduction of a tetrachloroaurate ion [AuCl_4_]^−^ occurs at a potential of E_0_ = +1.0 V by the hydrogen electrode (+ 0.78 V by the silver chloride electrode) according to the reaction:

[AuCl_4_]^−^ + 3e^−^ = Au_0_ + 4Cl^−^ (3)

**Figure 4:**
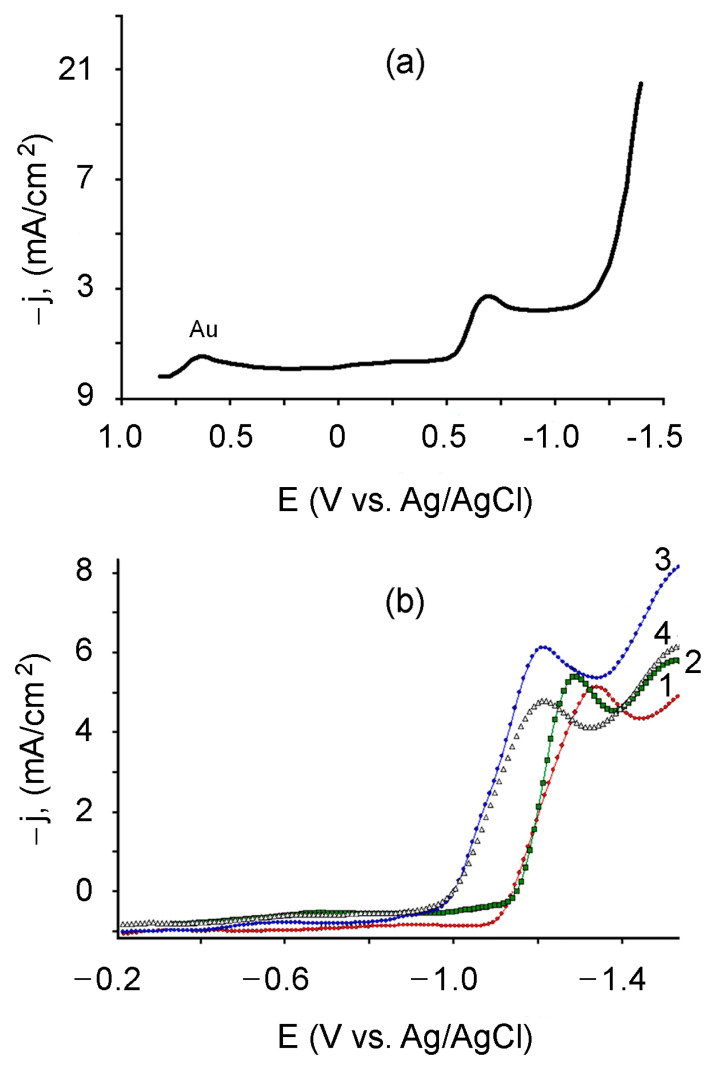
Polarization curves recorded in: (**a**)—5 mmol/L HAuCl_4_/0.1 mol/L LiClO_4_, (**b**)—electrolyte 1 (1), electrolyte 2 (2), electrolyte 3 (3), electrolyte 4 (4). The potential sweep rate is 10 mV/s.

The experimental results obtained showed that the electroreduction of gold on the steel cathode initiated at E = + 0.78 V with a maximum at E = + 0.65 V, relatively to the silver chloride electrode (Figure 4a).

On the other hand, as follows from Figure 4b (curves 1 and 2), the potential for electrochemical initiation of acrylamide polymerization and the initiation of the PMAA film formation falls in the cathode region and is E_i_ ≥ −1.1 V. Here, the optimal potential values for the formation of the PMAA film from the base electrolyte _1_ are in the range (−1.16 ÷ −1.20) V [32,34]. The introduction of HAuCl_4_ to the monomer composition leads to a shift in the potential of the beginning of the formation of the PMAA film towards a decrease in the cathode potential to E = −0.95 V (Figure 4b, curves 3 and 4). These results indicate that, in the range of potentials (−0.95 ÷ −1.20) V, simultaneous electrolytic reduction of Au^0^ and electrochemical initiation of the polymerization of acrylamide with the formation of a PMAA film is possible.

**Figure 5:**
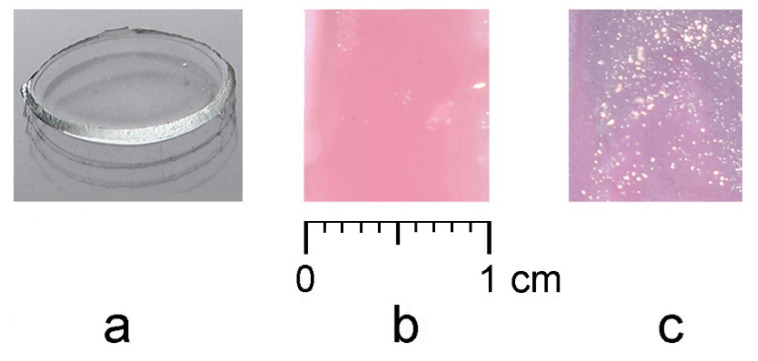
Polymer films formed from the electrolyte 1 (**a**), electrolyte 3 (**b**), and electrolyte 4 (**c**).

The polymer films prepared by electropolymerization from the base electrolytes 1 and 2 represent an elastic, colorless, and transparent material in the swollen state in water (Figure 5a). On the other hand, the color of the films electrosynthesized in the presence of HAuCl_4_ (electrolytes 3 and 4) acquires a pink-lilac color (Figure 5b,c).

The color intensity of the composite film depends on the concentration of HAuCl_4_ in the electrolytes and the amount of electroreduced gold in the film and is enhanced in the presence of chitosan in the electrolyte. In the latter case, the color of the composite becomes lilac (Figure 5c). The change in the color of the film visually indicates the inclusion of gold in the polymer matrix and the formation of the PMAA/AuNPs composite.

**Figure 6:**
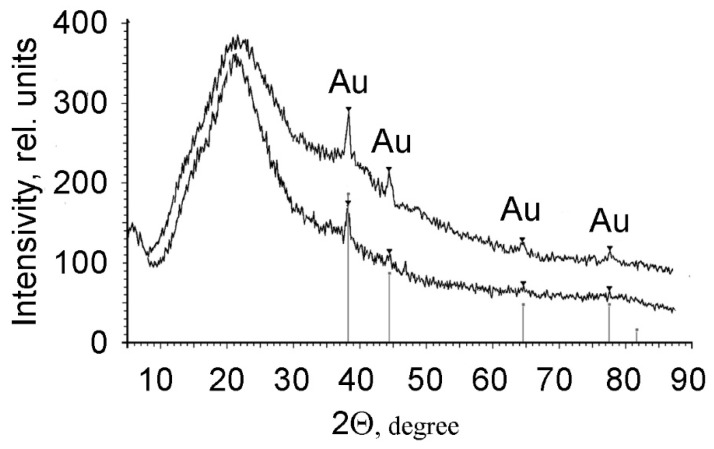
X-ray diffraction patterns of PMAA/AuNPs of composites formed from electrolytes 3 (1) and 4 (2).

The inclusion of metallic gold in the polymer film is also corroborated by the results of X-ray diffraction analysis. As follows from Figure 6, the composite PMAA/AuNPs films comprise an amorphous matrix with the inclusion of crystal particles of Au^0^, the presence of which is indicated by a series of bands corresponding to gold.

**Figure 7:**
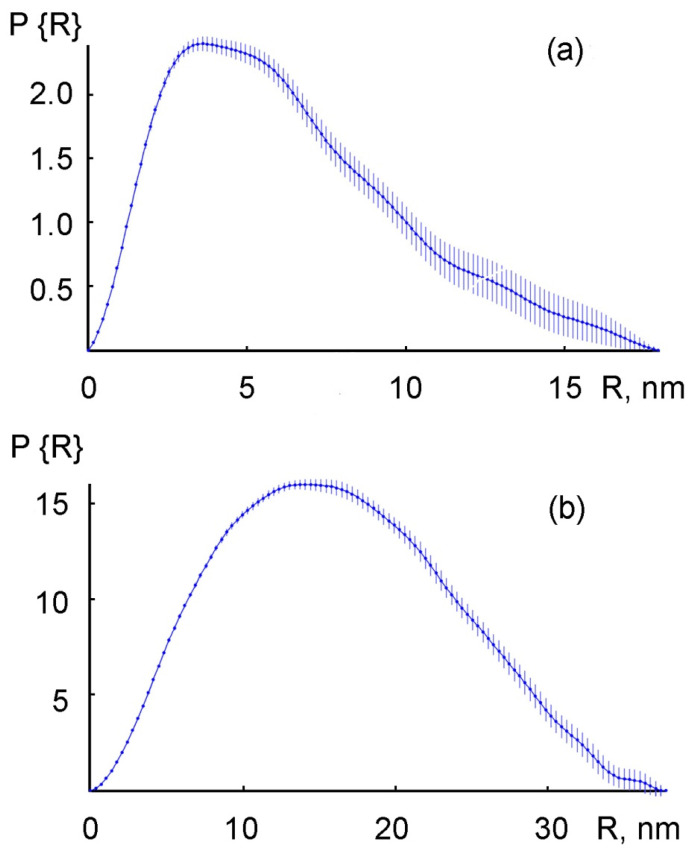
SAXS spectra of AuNPs size distribution for composites formed from electrolyte 3 (**a**) and electrolyte 4 (**b**).

The size of the AuNPs particles immobilized in the PMAA/AuNPs composite film is determined by the small-angle X-ray scattering (SAXS) (Figure 7).

As can be seen from Figure 7, the AuNPs electroreduced from the base electrolytes with the addition of HAuCl_4_ have a wide range of radii distribution. The radius of the particles depends on the composition of the electrolyte and the concentration of the components. Here, in the composite formed from electrolyte _3_, the maximum number of particles has a radius of 3.5–5.0 nm (Figure 7a). Here, the average radius of the particles increases along with HAuCl_4_ concentration growing (Table 2).

**Figure 8:**
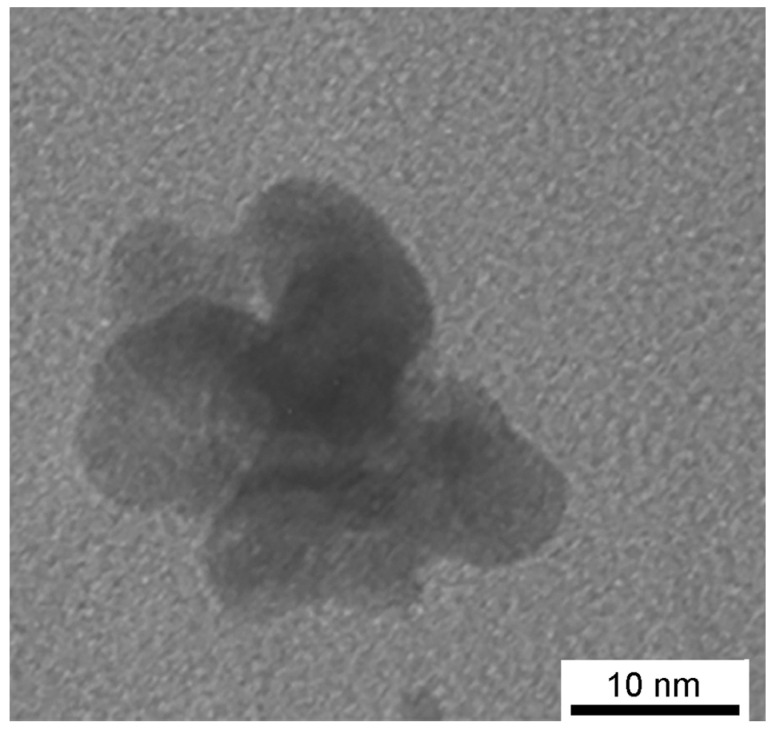
Micrographs of gold nanoparticles in PMAA/AuNPs composite film. Electrolyte 3. Libra-200FE transmission electron microscope, Carl Zeiss (Germany).

It was found that, at a concentration of C_(HAuCl4)_ = 1 mmol/L, Au_0_ particles with an average radius of ~5.0 nm predominate, whereas at C_(HAuCl4)_ = 3 mmol/L, their size reaches 7.0 nm. The introduction of chitosan into the monomer composition (electrolyte 4) increases the radius of the AuNPs nanoparticles up to 14.0–17.0 nm (Figure 7b). The microscopic studies showed that the AuNPs had a spherical shape and could combine into larger clusters containing up to 10 or more particles (Figure 8).

The analysis of the experimental data enables one to conclude that gold nanoparticles are formed and embedded in the polymer film directly during the electrolysis process. Here, at the cathodic potential E ≥ −0.95 V, the reduction of [AuCl_4_]^−^ ions to Au^0^, the electrochemical initiation of monomer polymerization, and the growth of the polymer matrix occur simultaneously, resulting in the formation of the PMAA/AuNPs composite. It should be mentioned that the formation of the PMAA/AuNPs composite is more energetically advantageous (Figure 4b, curves 3 and 4) as compared to the synthesis of the PMAA base film (Figure 4b, curves 1 and 2), since it proceeds at a lower cathode potential.

Thus, the technology we developed for the production of the PMAA/AuNPs composite combines all the intermediate stages in one cycle, which significantly simplifies the process and reduces the total synthesis time to 3–10 min.

### 3.2. Dynamics of the Formation of PMAA Films and PMAA/AuNPs Composite

**Figure 9:**
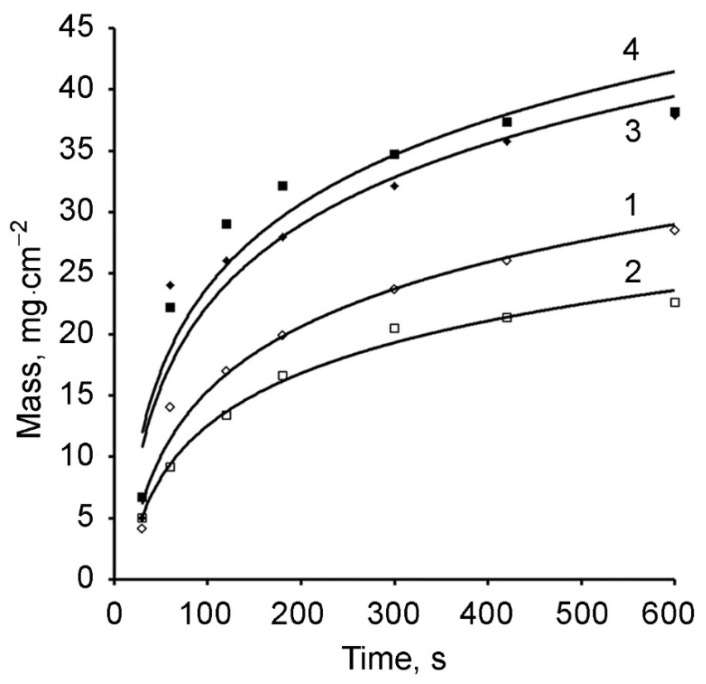
Dependence of the polymer mass on the electropolymerization time: electrolyte 1 (1), electrolyte 2 (2), electrolyte 3 (3), electrolyte 4 (4).

The dynamics of the formation of polymethylolacrylamide films and PMAA/AuNPs composites with the inclusion of gold nanoparticles in the polymer is shown in Figure 9.

**Figure 10:**
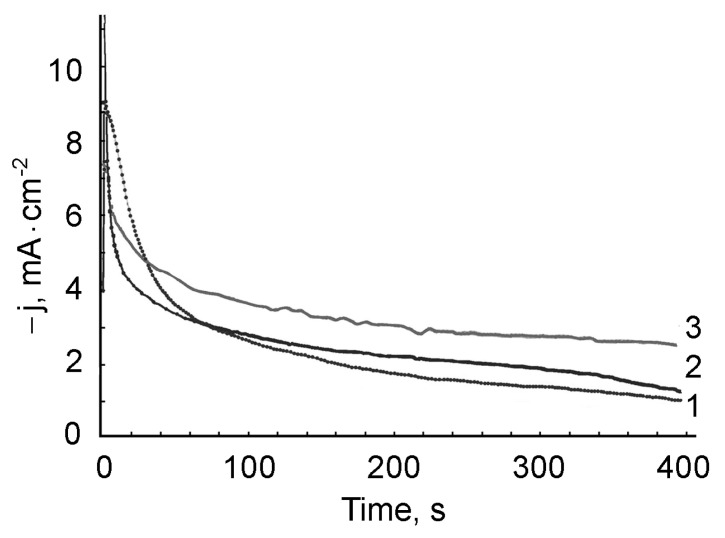
Current density versus electropolymerization time and electrolyte composition: electrolyte 1 (1), electrolyte 2 (2), electrolyte 4 (3). E = −1.2 V.

For all the studied electrolytes, the masses of the PMAA films (Figure 9 curves 1 and 2) and the PMAA/AuNPs composites (Figure 9, curves 3 and 4) depend on the synthesis time. The rate of the mass gain is maximal during the first 60 s of electrolysis, then the growth dynamic decreases. This course of the process is determined by both an increase in the thickness of the weakly conducting polymer layer on the cathode surface and the associated diffusion restrictions of the electrolyte supply to the electrode surface and a drop in the current density (Figure 10), and a decrease in the rate of electropolymerization.

The immobilization of gold nanoparticles into the PMAA matrix results in an increase of the total mass of the composite (Figure 9, curves 3 and 4) as compared to films without gold (Figure 9, curves 1 and 2). At a synthesis time of 60 s, this difference reaches 10–15 mg/cm^2^, which is 26–40% of the composite mass (Table 3).

It was found that the inclusion of gold nanoparticles into the polymer matrix affected the electrical conductivity of the coating formed at the cathode. As can be seen from Figure 10, for all the electrolytes during the electropolymerization, as the polymer layer is formed, the current density decreases due to the overlap of the cathode with a layer of a weakly conducting phase. Here, the lowest residual current (*i*_res_) is observed for the PMAA coatings formed from the base electrolytes 1 and 2 (Figure 10, curves 1, 2). On the other hand, the inclusion of AuNPs metal particles in the polymer leads to an increase in *i*_res_, i.e., to an increase in the electrical conductivity of the composite PMAA/AuNPs (Figure 10, curve 3).

### 3.3. Structure, Morphology and Properties of PMAA/AuNPs Composites

As was shown in [38], the electrosynthesized PMAA films had a porous structure and swelled in water. In the present work, it was found that the inclusion of AuNPs in the PMAA film slightly reduced the swelling capacity of *S_sw_* of the composite. However, the immobilization of gold nanoparticles in the PMAA matrix makes the composite structure more rigid, as a result of which the linear expansion coefficient *L*_1_ of the composite material decreases during the swelling, whereas the linear compression coefficient *L*_2_ increases in the course of drying of the films (Table 4).

**Figure 11:**
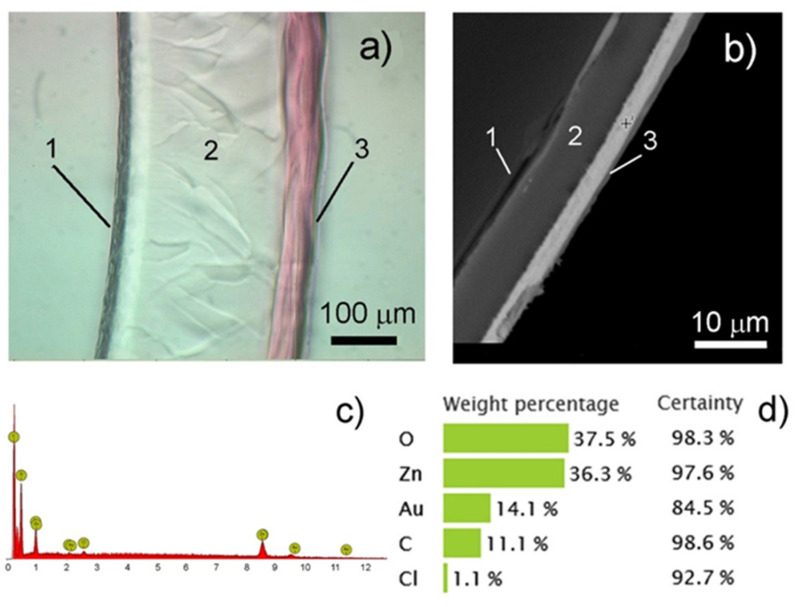
Cross section (**a**,**b**) and elemental composition (**c**,**d**) of the PMAA/AuNPs composite film. Electrolyte 3. Swollen state (**a**), dry state (**b**). Optical microscope Axioplan 2 Imaging, Carl Zeiss with a photographic attachment Panasonic NV-GS330EE-S and electron microscope PHENOM pro-X.

The microscopic examinations of thin cross-sections of the composites PMAA/AuNPs showed that the film had a complex asymmetric structure over the layer thickness. On the cross-section of the film, three layers are clearly visible: the near-electrode layer 1, the middle layer 2, and the layer 3 formed at the film/solution interface (surface layer) (Figure 11a). The thickness of the layers in the dry (b) and swollen (a) state is, respectively: 1–1.5/15–20 μm (1), 6–7/230–232 μm (2), 2–3/66–68 μm (3).

**Figure 12:**
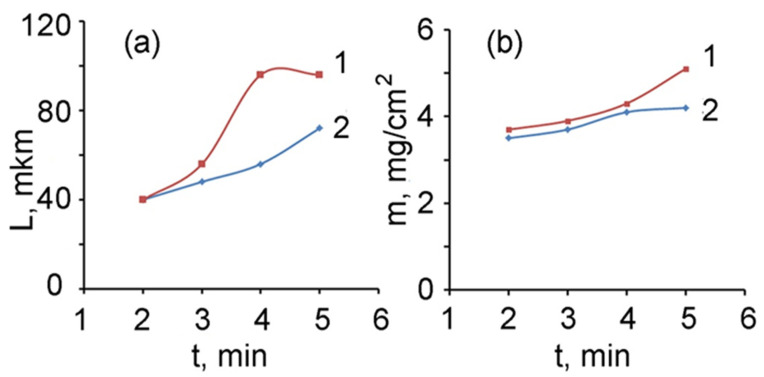
Thickness of layer 3 (**a**) and specific gravity of the PMAA/AuNPs composite (**b**) versus electrolysis time. 1–electrolyte 4, 2–electrolyte 3.

The electroreduced gold nanoparticles are mainly concentrated in layer 3 (Figure 11a), which is located on the side of the film facing the solution during the synthesis and is most accessible for saturating the film with a gold-containing electrolyte. The results shown in Figure 12 are directly related to the structural features of the composite and the predominant immobilization of AuNPs in layer 3. The thickness of layer 3 increases along with the increase in electrolysis time (Figure 12a). In this case, the amount of electroreduced gold in this layer and, accordingly, the total mass of the composite film increases. Similarly, the specific gravity of the forming composite material increases, in which the proportion of metal AuNPs increases (Figure 12b).

### 3.4. Sensor Properties of the Composite PMAA/AuNPs

Despite the widespread use of electrochemical biosensors, they have a number of disadvantages that limit their practical application. These disadvantages include the difficulty of immobilizing and maintaining the stability of an enzyme. As a result, there is an active search for new highly sensitive, selective, and express sensors that exclude the use of enzymes. Recently, enzyme-free electrochemical sensors based on nanomaterials have been of great interest. Such sensors are extensively used to determine hydrogen peroxide, which is a product or reagent in most reactions occurring in living and plant cells [39,40,41,42]. The enzyme-free determination of hydrogen peroxide catalyzed by nanomaterials is carried out on the basis of reactions of reduction or oxidation of hydrogen peroxide on an electrode.

**Figure 13:**
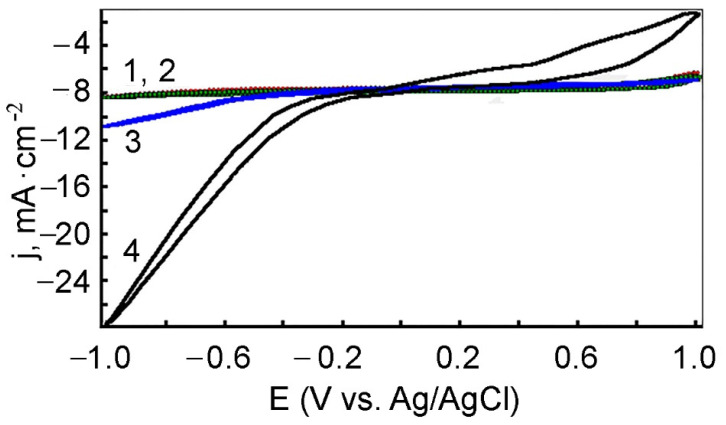
Cyclic voltammograms for electrodes modified with PMAA and PMAA/AuNPs in PBS (1, 2) and in PBS with 0.08 mol/L H_2_O_2_ (3, 4). Scan rate 100 mV/s.

The electrodes modified with PMAA and PMAA/AuNPs films were used to study the electrocatalytic activity (sensory sensitivity) of the electrosynthesized PMAA/AuNPs composite towards hydrogen peroxide. Cyclic voltammetric studies showed that both the initial PMAA polymer film (Figure 13, curve 1) and the PMAA/AuNPs composite (Figure 13, curve 2) were electrochemically stable over a wide range of potentials from +1 V to −1 V.

The addition of 0.08 mol/L of H_2_O_2_ to the phosphate buffer solution (PBS, pH 6.86) results in a noticeable jump in the potential in the range of H_2_O_2_ reduction (Figure 12). Here, the current density at the maximum point for the PMAA/AuNPs composite containing gold nanoparticles (Figure 13, curve 4) is 2.7 times higher than for the pure PMAA (Figure 13, curve 3). Consequently, the electrodes modified with the composite film PMAA/AuNPs exhibit selective behavior towards hydrogen peroxide.

**Figure 14:**
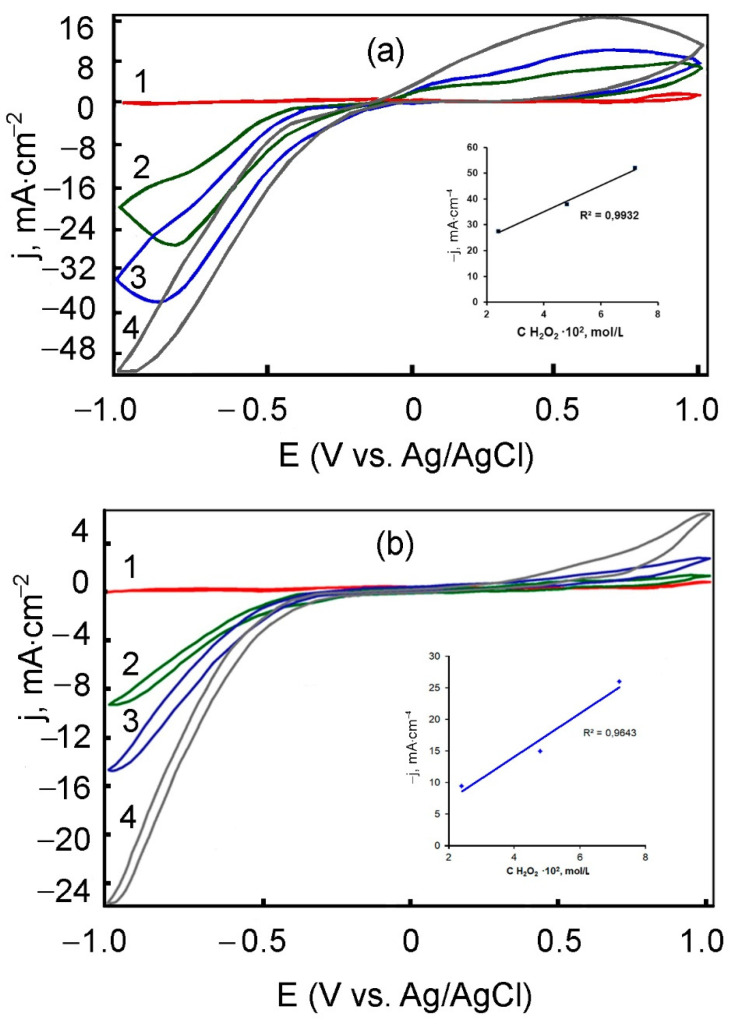
Cyclic voltammograms and dependence of the current density of the hydrogen peroxide reduction peak on its concentration for electrodes with PMAA/AuNPs (electrolyte 3) (**a**) and PMAA/AuNPs (electrolyte 4) (**b**) films in H_2_O_2_/PBS. H_2_O_2_ concentration (mol/L): 0 (1), 2.4 × 10^−2^ (2), 4.8 × 10^−2^ (3), 7.2 × 10^−2^ (4). Scan rate 100 mV/s.

The dependence of the rate of reduction of hydrogen peroxide on its concentration on the electrodes modified with films with the addition of AuNPs was studied by cyclic voltammetry (Figure 14).

Sequential addition of 2.4 × 10^−2^ mol/L H_2_O_2_ in PBS leads to a sharp increase of the current density at E = −0.85 ± −0.95 V (potential of hydrogen peroxide electroreduction) (Figure 14). Moreover, the highest rate of reduction of hydrogen peroxide and higher sensitivity of the modified electrode (maximum current density) were observed for the composite formed from the electrolyte _3_ without chitosan (Figure 14a). This is presumably due to a looser structure of such a composite and a lighter diffusion permeability of the substance.

It was established that the current density at the maximum point was proportional to the concentration of H_2_O_2_. Here, the confidence coefficient of the approximation is higher for the electrode modified with the PMAA/AuNPs film formed from electrolyte 3 (Figure 14a) rather than for the electrolyte 4 (Figure 14b), which includes chitosan.

**Figure 15:**
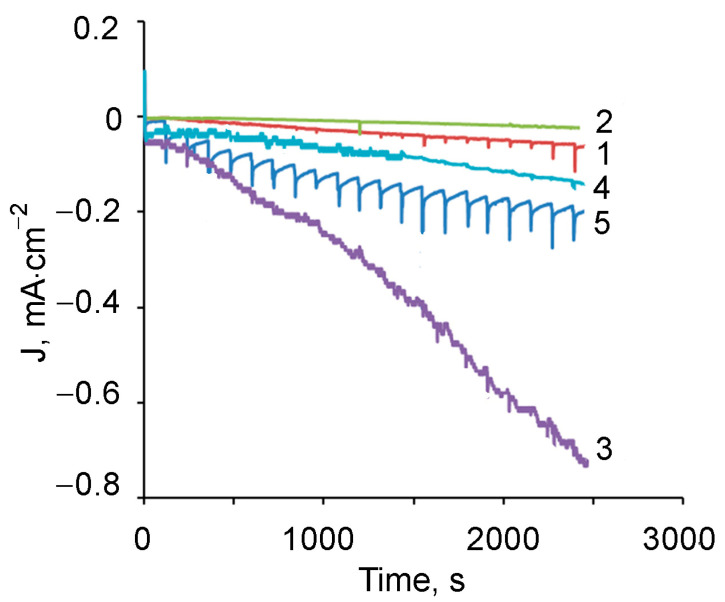
Chronoamperometric dependences for electrodes: SS/PMAA1 (1), SS/PMAA2 (2), SS/PMAA3/AuNPs (3), SS/PMAA4/AuNPs (4) and SS (5). PBS (pH 6.68). E = −0.35 V. The sequential adding 1.95 mmol/L H_2_O_2_ at 1-min intervals.

Chronoamperometric measurements were performed on pure stainless steel SS and composite electrodes SS/PMAA1 (electrolyte 1) and SS/PMAA2 (electrolyte 2), as well as the composite electrodes with gold nanoparticles SS/PMAA3)/AuNPs (electrolyte 3) and SS/PMAA4/AuNPs (electrolyte 4). H_2_O_2_ was sequentially added at 1.95 mmol/L in PBS with an interval of 1 min at electrode polarization E = −0.35 V (Figure 15).

A comparison of the chronoamperometric signals for the sequential introduction of H_2_O_2_ showed that the electrodes modified with films without the inclusion of gold virtually did not react to hydrogen peroxide (Figure 15, curves 1, 2). The sensitivity of SS/PMAA4/AuNPs composite is not much higher due to high density and insulating effect of the chitosan-containing films (Figure 15, curve 4). A good linear response to H_2_O_2_ from 0.1 mmol/L to 10 mmol/L is observed on the stainless steel electrode (Figure 15, curve 5). However, the maximum response to hydrogen peroxide is observed on the electrode modified with SS/PMAA3/AuNPs (Figure 15, curve 3), also with much higher sensitivity and stable signal than on the stainless steel.

The results obtained enable us to conclude that the composite electrode modified with PMAA3/AuNPs film has excellent electrocatalytic characteristics towards H_2_O_2_ and can be promising for use in electrochemical sensors, as well as a sensor for hydrogen peroxide.

## 4. Conclusions

The possibility of electrochemical formation of the composite polymethylolacrylamide film/gold nanoparticles has been investigated. It has been demonstrated that the method of electropolymerization of aqueous solutions based on acrylamide and formaldehyde in the presence of HAuCl_4_ allows electrochemically reducing nanoscale gold particles and immobilizing them into a growing polymer film. The special feature of this approach consists in the fact that the process is performed in a single stage, when the non-conducting (dielectric) polymer matrix is formed simultaneously with gold being reduced in the form of nanoparticles, which are captured by the polymer film and stabilized in it. It was found that the formation of the PMAA/AuNP composite is energetically more favorable than the synthesis of the main PMAA film, since it proceeds at a lower cathodic potential. Here, the total time of the composite formation does not exceed five minutes. The inclusion of AuNPs in the polymethylolacrylamide film has been corroborated visually, as well as by X-ray phase analysis, small-angle X-ray scattering, microscopy, and element analysis. The spherical shape and radius (3.5–14 nm) of gold nanoparticles have been determined. The AuNPs particles can combine into larger clusters.

The dynamics of the formation of the PMAA/AuNPs composite have been defined. An asymmetric structure of the composite film over the layer thickness was revealed. The gold nanoparticles are mainly concentrated in the near-electrode and near-solution layers of the composite. The gold content in the composite increases along with the increase of the concentration of HAuCl_4_ in the electrolyte. The electrocatalytic activity of the composite has been established. It is shown that the composite PMAA/AuNPs (electrolyte 3) has a good sensory sensitivity towards hydrogen peroxide.

## Figures and Tables

**Table 1:** Electrolyte compositions for electropolymerization.

Components	Concentration	Electrolyte			
		1	2	3	4
Acrylamide (AA)	3 mol/L	AA	AA	AA	AA
Formaldehyde (F)	3 mol/L	F	F	F	F
Zinc chloride	0.2 mol/L	ZnCl_2_	ZnCl_2_	ZnCl_2_	ZnCl_2_
N, N-methylene-bis-acrylamide (MBAA)	0.05 mol/L	MBAA	MBAA	MBAA	MBAA
Chitosan (Chs)	0.1%	-	Chs	-	Chs
Tetrachloroauric acid	1–4 mmol/L	-	-	HAuCl_4_	HAuCl_4_

**Table 2:** Dependence of the size of gold nanoparticles on the concentration of tetrachloroauric acid.

Concentranion (HAuCl_4_), mmmol/L	Average Radius AuNPs, nm
1	5.12 ± 0.30
2	5.78 ± 0.15
3	6.90 ± 0.47

**Table 3:** Dependence of the polymer weight on the time of composite formation.

Time, s	Polymer Mass, mg/cm^2^			
	Electrolyte 1	Electrolyte 2 2	Electrolyte 3 3	Electrolyte 4
30	4.10	-	4.99	6.70
60	14.07	9.20	24.02	22.21
120	17.00	13.40	-	-
180	19.90	16.65	27.97	32.15
300	23.67	20.50	32.12	33.67
600	28.48	22.60	37.88	38.20

**Table 4:** Swelling, linear elongation, and linear shortening coefficients of electrosynthesized PMAA/AuNPs films.

Composition	*m_sw_*, gm	*m_dry_*, gm	*S_sw_,* %	*Ø_in_*, mm	*Ø_sw_,* mm	*Ø_dry_*, mm	*L*_1_	*L*_2_
Electrolyte 1	0.0217	0.0044	393	8.5	12	7	1.41	1.21
Electrolyte 4	0.0129	0.0027	377	8.5	11	6	1.29	1.42

## Data Availability

Not applicable.
